# Inactivation of Antibiotic-Resistant Bacteria in Wastewater by Ozone-Based Advanced Water Treatment Processes

**DOI:** 10.3390/antibiotics11020210

**Published:** 2022-02-07

**Authors:** Takashi Azuma, Masaru Usui, Tetsuya Hayashi

**Affiliations:** 1 Department of Pharmacy, Osaka Medical and Pharmaceutical University, Takatsuki 569-1094, Japan; t.hayashi@soai.ac.jp; 2 Food Microbiology and Food Safety, Department of Health and Environmental Sciences, School of Veterinary Medicine, Rakuno Gakuen University, Ebetsu 069-8501, Japan; usuima@rakuno.ac.jp; 3 Department of Food and Nutrition Management Studies, Faculty of Human Development, Soai University, Suminoe-ku, Osaka 559-0033, Japan

**Keywords:** antimicrobial resistance (AMR), inactivation, ozonation, advanced oxidation process (AOPs), sewage treatment plant (STP), river environment

## Abstract

The inactivating effect of ozone (O_3_)-based advanced oxidation processes (AOPs) (O_3_/H_2_O_2_, O_3_/UV, and O_3_/UV/H_2_O_2_ systems) on antimicrobial-resistant bacteria (AMRB) and antimicrobial-susceptible bacteria (AMSB) in sewage treatment plant (STP) wastewater was investigated. The AMRB were grouped into six classes: carbapenem-resistant *Enterobacteriaceae* (CRE), extended-spectrum *β*-lactamase (ESBL)-producing *Enterobacteriaceae* (ESBL-E), multidrug-resistant *Acinetobacter* (MDRA), multidrug-resistant *Pseudomonas aeruginosa* (MDRP), methicillin-resistant *Staphylococcus aureus* (MRSA), and vancomycin-resistant *Enterococcus* (VRE); these classes constituted the World Health Organization (WHO) global priority list of AMRB. The results indicate that O_3_-based advanced wastewater treatment inactivated all AMRB and AMSB (>99.9%) after 10 min of treatment, and significant differences (*p* < 0.5) were not observed in the disinfection of AMRB and AMSB by each treatment. Altered taxonomic diversity of micro-organisms based on 16S rRNA gene sequencing via O_3_/UV and O_3_/UV/H_2_O_2_ treatment showed that advanced wastewater treatments not only inactivated AMRB but also removed antimicrobial resistance genes (AMRGs) in the wastewater. Consequently, this study recommends the use of advanced wastewater treatments for treating the STP effluent, reducing environmental pollution, and alleviating the potential hazard to human health caused by AMRB, AMSB, and infectious diseases. Overall, this study provides a new method for assessing environmental risks associated with the spread of AMRB and AMSB in aquatic environments, while keeping the water environment safe and maintaining human health.

## 1. Introduction

The emergence and spread of antimicrobial-resistant bacteria (AMRB) has become a serious situation in clinical sites worldwide [1,2,3,4]. AMRB are widely detected in the aquatic environment [5,6,7,8,9]. The spread of AMRB not only makes it difficult to treat infectious diseases but also increases the risk of epidemics and aggravation. Taking effective measures to prevent the prevalence of AMRB has become urgent [10,11,12]. The connection between humans and the environment continues to complicate. With AMRB flowing into the aquatic environment, both direct infections associated with drinking and swimming and indirect infections through animals, agriculture, and fisheries are of concern [9,13,14,15,16,17]. The World Health Organization (WHO) launched a Global Action Plan on Antimicrobial Resistance (AMR) based on the One Health approach. The goal of the plan is to provide optimal health for people, animals (domestic and wild), and the environment by considering interactions between all three systems [18,19,20]. This plan also requires every country to institute its own national action plan by performing extensive research on the occurrence, environmental fate, and risk assessment of AMRB flowing into water bodies [21]. Japan has implemented the Action Plan on Antimicrobial Resistance to prevent their prevalence [22,23,24].

Previous studies have reported that the effluent from sewage treatment plants (STPs) is one of the main sources of river pollution in urban areas, where the population is concentrated and the sewerage coverage is highly developed [25,26,27,28]. Some AMRB flowed into the river environment without being sufficiently inactivated by conventional disinfection processes, such as chlorine [27,29], which is widely used as a disinfection treatment in STPs worldwide [5,30]. In addition, various wastewater antimicrobials were also difficult to completely remove by the conventional sewage treatment process and entered the river environment. These residual antimicrobials have toxic effects on the aquatic ecosystem and become potential factors that promote the formation of new AMRB in the aquatic environment [31,32]. Therefore, evaluating the effectiveness of the advanced oxidation processes (AOPs) is becoming increasingly important for reducing the pollution load into rivers and creating environmental risks [33,34,35].

Several recent studies have evaluated the inactivation of AMRB in water using various disinfection methods, including the use of Fenton [36,37], ultrasonication [38], electrolysis [39], TiO_2_ [40], persulfate [41], graphitic carbon nitride (g-C_3_N_4_) [42], UV/chlorine [43], and ozone (O_3_) [37,44]. Among these, O_3_ has a high oxidizing power (oxidation potential 2.1 V) [45] and is effective for decolorization, deodorization, sterilization, etc. [46]. It has been shown to be effective not only for inactivating pathogenic microorganisms [47,48] but also for removing micropollutants such as pharmaceutical residues and endocrine disruptors in wastewater [49,50]. In addition, by using O_3_ and hydrogen peroxide (H_2_O_2_) together (O_3_/H_2_O_2_) or O_3_ and ultraviolet rays (O_3_/UV), hydroxyl radical (OH) [51] exhibits a stronger oxidizing power (oxidation potential 2.8 V) than that obtained using O_3_ alone; thus, it is possible to improve the efficiency of O_3_ treatment [52,53,54]. Nevertheless, research on the inactivation of AMRB and the development of countermeasures to reduce their impact remains limited [55,56]. Therefore, it seems necessary to investigate the inactivation of AMRB in wastewater in detail for a practical and comprehensive understanding of the environmental risks of AMRB in rivers. Previous research reported the inactivation profiles of AMRB obtained by treatment with O_3_ alone using actual wastewater from sewage treatment plants [57,58]. Given this situation, the current study evaluated the effectiveness of O_3_/H_2_O_2_, O_3_/UV, and O_3_/UV/H_2_O_2_ systems for inactivation of a group of AMRB and antimicrobial-susceptible bacteria (AMSB) in STP effluent to better understand the environmental risk management of AMRB in the water environment. Evaluating the inactivation of AMRB in real wastewater samples via O_3_-based AOPs will provide useful insights into the effectiveness of the inactivating effect of wastewater treatment in overcoming the challenge of water contamination and pollution by AMR.

## 2. Materials and Methods

### 2.1. Microbes and Reagents

Six classes of AMRB were given global priority by the WHO [21,59] and are reported to be in the clinical range [3,60]—namely CRE, extended-spectrum *β*-lactamase (ESBL)-producing *Enterobacteriaceae* (ESBL-E), multidrug-resistant *Acinetobacter* (MDRA), multidrug-resistant *Pseudomonas aeruginosa* (MDRP), methicillin-resistant *Staphylococcus aureus* (MRSA), and vancomycin-resistant *Enterococcus* (VRE) were investigated.

The AMRB prevalence was analysed by screening microbes grown on different chromogenic agar methods: chromID CARBA (bioMérieux S.A., Marcy-l’Étoile, France) for detection of CRE, chromID ESBL for ESBL-E, chromID MRSA for MRSA, chromID VRE New for VRE, CHROMagar MDRA for MDRA, and CHROMagar MDRP for MDRP (Kanto Chemical Co., Inc., Tokyo, Japan). Similarly, AMSB was analysed by screening microbes grown on different chromogenic agar methods without antimicrobial agents: CHROMagar Acinetobacter for *Acinetobacter*, chromID S. aureus Elite for *Staphylococcus aureus* (*S*. *aureus*), chromID CPS Elite for *Enterococcus*, PASA medium (Nippon Becton Dickinson Company, Ltd., Tokyo, Japan) for *Pseudomonas aeruginosa* (*P. aeruginosa*), and XM-G agar (Nissui Pharmaceutical Co., Ltd., Tokyo, Japan) for *Escherichia coli* (*E. coli*). Ultra-pure Milli-Q water (18.2 MΩ·cm; MilliporeSigma, Watford, UK) with pH adjusted to 7.0 and 10 M sterilized phosphate buffer were used for dilution. Analytical grade hydrogen peroxide (30%) and sodium thiosulfate (>98%) were purchased from FUJIFILM Wako Pure Chemical Corporation (Osaka, Japan).

### 2.2. Sampling

Samples were collected from an STP located in an urban area of Japan, as described previously [61]. The STP treats municipal sewage generated by a population of 420,000 individuals. The STP influent was first treated with conventional activated sludge and step anoxic/oxic treatment and discharged as the STP secondary effluent. The STP secondary effluent was treated with chlorine (contact with 0.9 mg NaClO/L for 15 min) for disinfection and discharged as the STP effluent. Three water types were collected from the STP: STP influent, STP secondary effluent, and STP effluent. Samples were collected in December 2018 on rain-free days when the recorded rainfall was >1 mm for the preceding two days [62]. The annual average chemical oxygen demand was 91, 19, and 17 mg/L for the STP influent, STP secondary effluent after biological treatment, and STP effluent, respectively. A stainless-steel pail sampler was used to collect wastewater samples, which were then placed in separate sterilized glass bottles. Sodium thiosulfate (0.5 g/L) was immediately added to each bottle for quenching residual chlorine [63,64]. Composite samplers could not be installed to sample the STP wastewater. Therefore, identical manual sampling at a fixed sampling frequency was used. All samples were immediately transported to the laboratory in a cooler box (within 2 h), stored in dark at 4 °C, and processed within 12 h.

### 2.3. Analytical Procedures

The number of each type of AMRB and AMSB in the samples was estimated following the protocols given by the manufacturers of growth mediums using previously described methods [65,66,67,68]. From every water sample, 1 mL was taken out and spread on separate agar plates and incubated at 37 ± 1 °C for 24 h in the dark. Then, the bacterial species were differentiated by the colour and morphology of the colonies [69,70,71]. The colonies were counted and the number of bacteria formed was expressed as colony-forming units per mL (CFU/mL). If the mean CFU was a whole number, the values were expressed as the nearest integer after applying the rounding off rule and counted as N.D. (not detected) if the values were <1. The relative reproducibility values (n = 3) for the AMRB (CRE, ESBL, MDRA, MDRP, MRSA, and VRE) were 15%, 27%, 18%, 12%, 20%, and 13%, respectively; those of AMSB (*Acinetobacter*, *Enterococcus*, *E. coli*, *P. aeruginosa*, *S. aureus*) were 9%, 13%, 15%, 7%, and 18%, respectively.

### 2.4. Inactivation of AMRB and AMSB by O_3_-Based AOPs

Inactivation of AMRB and AMSB by O_3_-based AOPs was investigated in a semi-batch reactor with an interior diameter of 10 cm and a height of 30 cm (effective volume of 2.2 L; Supplementary Materials Figure S1) [50]. The temperature of the test water was maintained at 20 °C through an external jacket by a thermostat water circulator (CTR-320, AGC Techno Glass Co. Ltd., Tokyo, Japan). The test water was agitated continuously at 300 rpm with a magnetic stirrer (SRS710DA, Advantec Toyo Kaisha, Ltd., Tokyo, Japan). Preliminary experiments indicated that a mixture of different water samples is appropriate for the performance of model laboratory experiments to determine the feasibility of the present method. STP secondary effluent and STP influent were mixed at a ratio of 9:1 (*v*/*v*) as a model sample (STP wastewater) for evaluating the inactivation of AMRB by O_3_-based AOPs, in accordance with a previous report [29,57,61] and the results in Section 3.1.

O_3_ was generated by an O_3_ generator (ED-OG-R6, Ecodesign Inc., Saitama, Japan) and injected into the reactor at a flow rate of 0.3 L/min with a concentration of 6.8 mg/L, which corresponded to a feed rate of 1.2 mg/L/min. This feed rate is similar to that used in a previous research on the effectiveness of O_3_ treatment on a wide range of microbes [63,72,73] and micropollutants [74,75]. This feed rate was similar to that used in STPs (7 ± 7 mg/L for 15 ± 5 min) [76]. UV irradiation was supplied by a 9 W low-pressure mercury lamp (TCGU60-250ZP, Miyakawa Corp., Tokyo, Japan) with a peak wavelength of 254 nm and an intensity of 2.8 mW/cm^2^, as reported in previous research [77,78]. The UV lamp was introduced into the reactor and kept separate from the water by using a quartz jacket. The initial H_2_O_2_ concentration was set at 5 mg/L (as effective concentration), as suggested in previous research [79,80].

The experiments were started by sparging O_3_ gas continuously into the filled reactor. A portion of the reactor solution (20 mL) was sampled at 0, 0.5, 1, 2, 5, and 10 min after the experiment started. These durations were determined by using the average contact times in Japanese STPs, which implement ozonation before discharging the effluent into rivers [76], and by previously reported values [74,81]. The O_3_ consumption was calculated from the balance in gas and liquid phases during the experiment [82,83]. Sodium thiosulfate was immediately added to each sample at a concentration of 1.0 g/L to mitigate the effects of any residual O_3_ and H_2_O_2_ and to quench reactive oxygen species such as hydroxyl radicals [63,64]. The samples were then stored at 4 °C in dark and processed within 12 h. Bacterial numbers and species survived from the treatment were analysed with differences in colony colour and morphology, as described in Section 2.3.

### 2.5. Bacterial Community Structure Analysis

Genomic DNA was extracted from the water samples using an Extrap Soil DNA Kit Plus v.2 (Nippon Steel Eco-Tech Corporation, Tokyo, Japan). The concentrations and purifications of DNA were determined by a Qubit^®^ 3.0 fluorometer (Thermo Fisher Scientific, Waltham, MA, USA) using a Qubit^®^ dsDNA BR Assay Kit (Thermo Fisher Scientific, Waltham, MA, USA) [84]. The V1–V2 region of the 16S ribosomal RNA (rRNA) gene of bacteria was used to characterise the bacterial communities [85,86]. For PCR amplification, the universal bacterial primers 27F/338R [87,88] were used. PCR was carried out in a T100 Thermal Cycler (Bio-Rad Laboratories, Inc., Hercules, CA, USA) in accordance with previous studies [89,90]. The PCR cycle consisted of a 3 min denaturation cycle at 95 °C, which was followed by 25 cycles at 95 °C for 30 s, 55 °C for 30 s, 72 °C for 30 s, and 72 °C for 5 min. Electrophoresis was conducted in 1.5% agarose gel using a Mupid-2plus System (Advance Co. Ltd., Tokyo, Japan) to examine the quality of PCR products [90], and the genes were sequenced on a MiSeq platform (Illumina Inc., San Diego, CA, USA) according to the manufacturer’s instructions and a previous report [57].

Sequence data were pre-processed and analysed in Flora Genesis software (Repertoire Genesis Inc., Osaka, Japan). Operational taxonomic units (OTUs) were picked by the open-reference method at a 97% identity level and annotated from the prefiltered Greengenes Database v.13.8 by the UCLUST algorithm [91,92]. Representative sequences of each OTU were extracted, and taxonomy was assigned by the Ribosomal Database Project classifier at a confidence threshold of 0.80 [93,94].

### 2.6. Statistical Analysis

The data of the tested traits were analysed by Microsoft Excel software. A paired *t*-test was performed to evaluate the difference in inactivation rates between AMRB and AMSB at *p* < 0.05 as statistical significance.

## 3. Results and Discussion

### 3.1. Occurrence of AMRB and AMSB in the STP Wastewater

Table 1 shows the occurrence of AMRB and AMSB in STP wastewater. All AMRB targeted in this study were detectable in the STP influent. The detection concentrations of AMRB ranged from 58 to 814 CFU/mL in the STP influent, N.D. to 201 CFU/mL in the STP secondary effluent, and N.D. to 34 CFU/mL in the STP effluent. These results show that AMRB were widely present in the wastewater, and they were almost removed in the wastewater treatment process of the STP; however, some of them (ESBL-E, MRSA, *Enterococcus*, and *Staphylococcus aureus*) were discharged into the river as effluent after chlorine disinfection treatment. These results were similar to those reported previously [71]. On the other hand, the number of AMSB ranged from 96 to 30,000 CFU/mL, N.D. to 836 CFU/mL, and N.D. to 219 CFU/mL for the STP influent, STP secondary effluent, and STP effluent, respectively. Inactivation of MRSA and *S. aureus* by chlorine disinfection was gradual, which may be attributed to stronger cell walls than those of other bacteria, which render them resistant to multiple environmental conditions [95,96].

### 3.2. O_3_-Based AOP Inactivation of AMRB and AMSB in STP Wastewater

**Figure 1:**
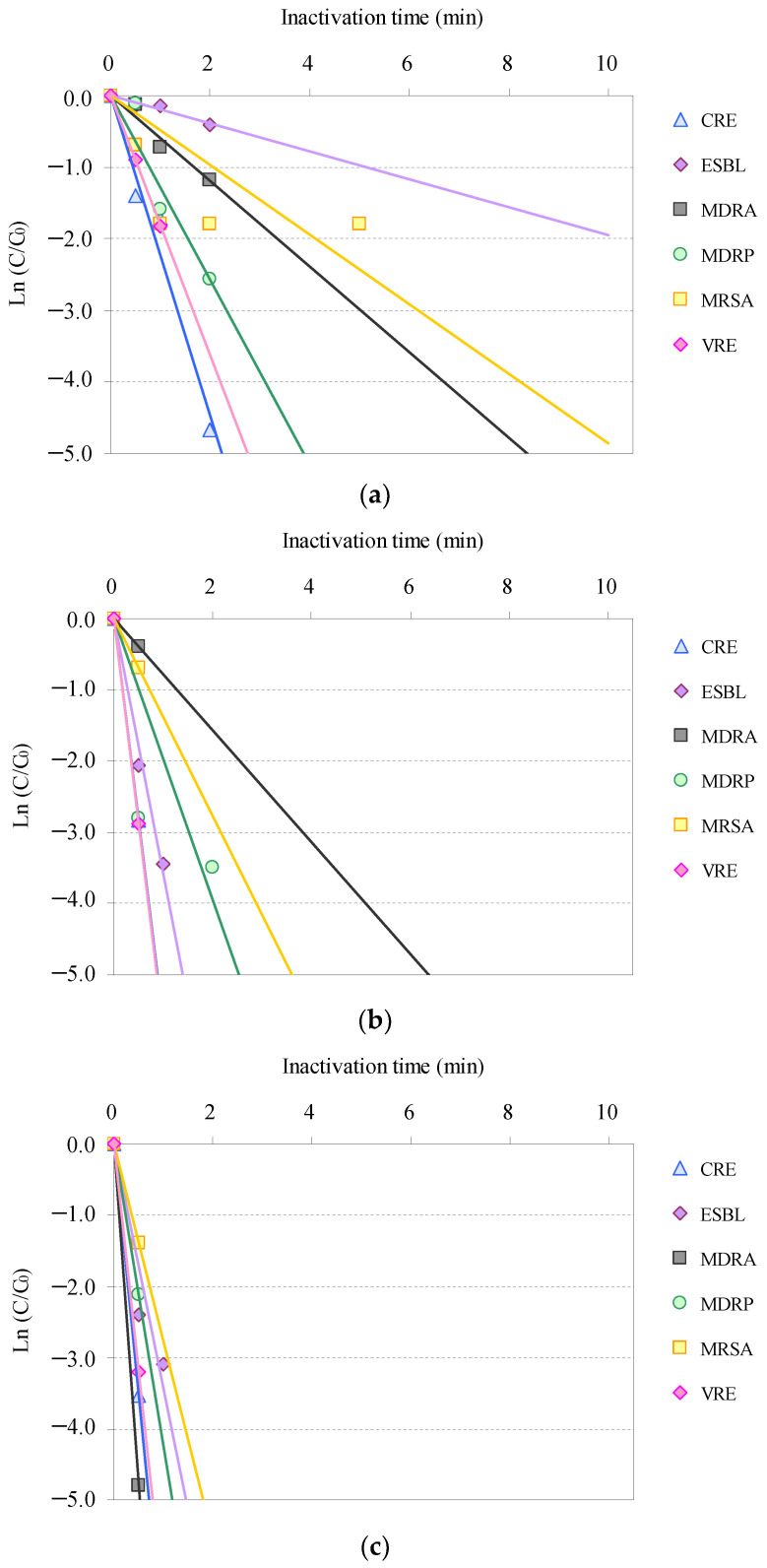
Relative residual antimicrobial-resistant bacteria (AMRB) under O_3_-based AOP treatment (C_0_: initial bacterial counts; C: bacterial counts after treatment): (**a**) O_3_/H_2_O_2_, (**b**) O_3_/UV, (**c**) O_3_/UV/H_2_O_2_. (CRE: carbapenem-resistant *Enterobacteriaceae*, ESBL-E: extended-spectrum *β*-lactamase-producing *Enterobacteriaceae*, MDRA: multidrug-resistant *Acinetobacter*, MDRP: multidrug-resistant *Pseudomonas aeruginosa*, MRSA: methicillin-resistant *Staphylococcus aureus*, and VRE: vancomycin-resistant *Enterococcus*).

**Figure 2:**
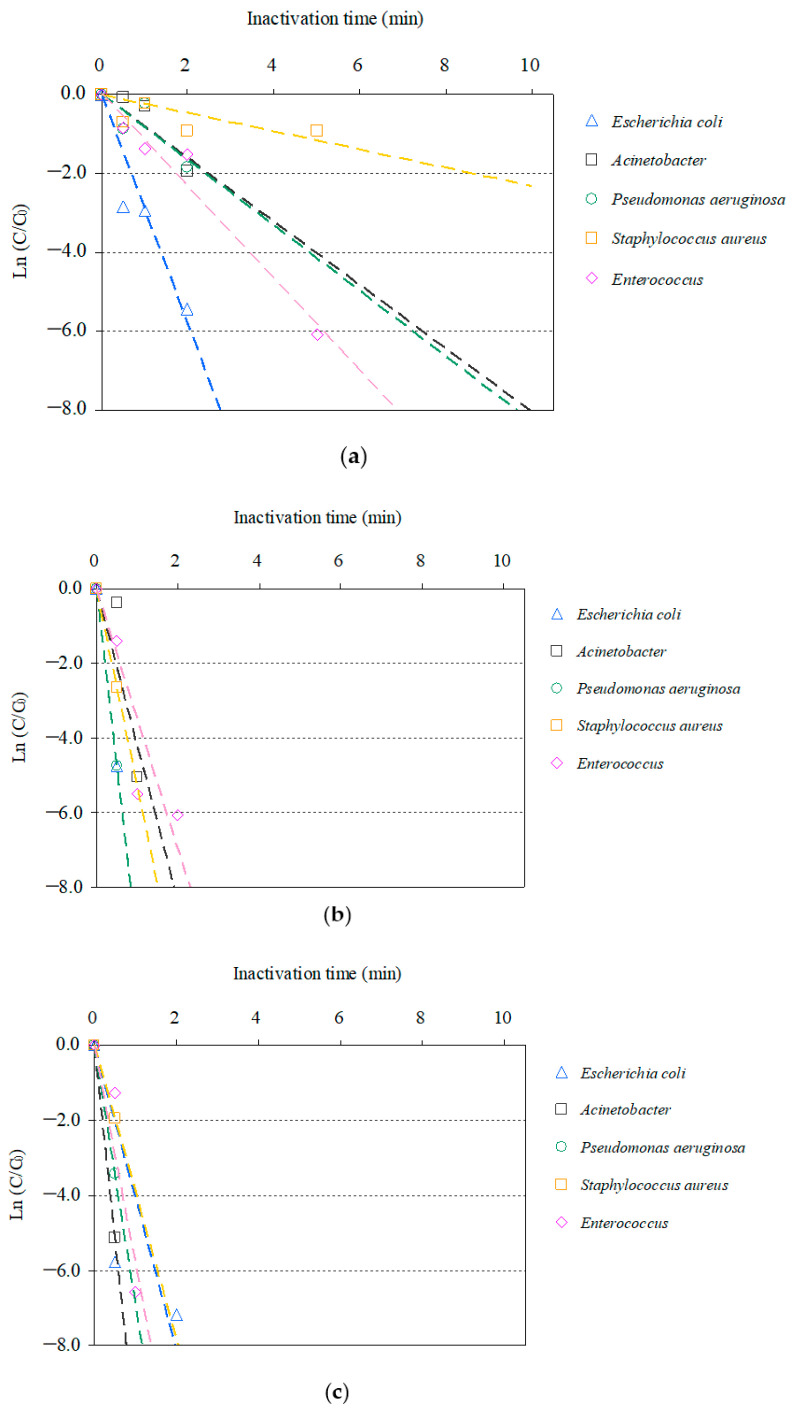
Relative residual antimicrobial-susceptible bacteria (AMSB) under O_3_-based AOPs treatment (C_0_: initial bacterial counts, C: bacterial counts after treatment): (**a**) O_3_/H_2_O_2_, (**b**) O_3_/UV, (**c**) O_3_/UV/H_2_O_2_.

Time-dependent inactivation profiles associated with the inactivation of the AMRB and AMSB in the STP wastewater via O_3_/H_2_O_2_, O_3_/UV, and O_3_/UV/H_2_O_2_ treatments are summarized in Figure 1 and Figure 2. Although the inactivation time differed among bacteria, all targeted AMRB and AMSB contained in the STP wastewater were inactivated by O_3_-based AOPs.

Inactivation of the AMRB and AMSB by O_3_-based AOPs followed pseudo-first-order kinetics, as previously reported for O_3_ disinfection of multiple bacteria and viruses [47,48,79,97]. CRE, MDRP, and VRE were rapidly inactivated >99% after 2 min; and ESBL-E and MDRA were inactivated >99% after 5 min in O_3_/H_2_O_2_ treatment. Meanwhile, MRSA was inactivated more gradually than other AMRB, with >99% inactivation after 10 min.

Similar profiles of inactivation for AMSB were observed: *E*. *coli* and *P*. *aeruginosa* were inactivated >99% after 2 min; and *Acinetobacter* and *Enterococcus* were inactivated >99% after 5–10 min. *S*. *aureus* was slowly inactivated with >99% inactivation after 10 min. In addition, no significant difference (*p* < 0.05) was observed in the effects of chlorination on AMRB and AMSB. These results are similar to those described in the previous section, supporting the effectiveness of the inactivation of AMRB in wastewater via ozonation.

Combined use of UV and O_3_ remarkably improved inactivation efficiencies. All targeted AMRB and AMSB were rapidly inactivated by O_3_/UV and O_3_/UV/H_2_O_2_ treatment. Within 1 min, >99% inactivation was completed for CRE, ESBL-E, MDRA, MDRP, MRSA, and VRE. In addition, synergistic improvement in inactivation was observed in O_3_/UV/H_2_O_2_ treatment. The inactivation rates after 0.5 min of treatment with O_3_/UV and O_3_/UV/H_2_O_2_ were 94% and 97% for CRE, 87% and 91% for ESBL-E, 32% and 99% for MDRA, 94% and 88% for MDRP, 50% and 75% for MRSA, and 94% and 96% for VRE, respectively. AMSB was also rapidly inactivated during both the treatments, and >99% inactivation was completed within 1 min for *Acinetobacter*, *Enterococcus*, *E. coli*, *P. aeruginosa*, and *S. aureus*. The improvement in the inactivation rate by the combined use of UV irradiation was related to the bactericidal activity of UV and the hydroxyl radicals generated as catalysts for O_3_ [47,79,98,99].

### 3.3. Distribution of the Inactivation Rate Constants of AMRB and AMSB by O_3_-Based AOP Treatment

The distribution inactivation rate constants for AMRB and AMSB in O_3_/H_2_O_2_, O_3_/UV, and O_3_/UV/H_2_O_2_ processes are summarized in Table 2.

The mean reaction rate constants for AMRB and AMSB were 1.1 ± 0.8 and 1.5 ± 1.0 min^−1^ for O_3_/H_2_O_2_, 3.2 ± 2.2 and 6.4 ± 3.0 min^−1^ for O_3_/UV, and 5.6 ± 2.6 and 6.2 ± 2.6 min^−1^ for O_3_/UV/H_2_O_2_, respectively. Interestingly, no significant differences were observed in the inactivation rates between AMRB and AMSB. Inactivation rate constants were improved by the combined use of UV irradiation with O_3_; the reaction rate constants of O_3_/UV and O_3_/UV/H_2_O_2_ treatments were significantly (*p* < 0.05) enhanced when compared with O_3_/H_2_O_2_ treatment for both AMRB and AMSB. Meanwhile, a statistically significant difference was not observed between the inactivation caused by O_3_/UV and O_3_/UV/H_2_O_2_ treatments. The estimated half-lives generally ranged from <0.1 to 1 min. The detailed distribution of half-lives for AMRB and AMSB are summarized in Table S1 (Supplementary Materials).

Previous research reported that the inactivation rate constants for AMRB and AMSB in wastewater subjected to O_3_ treatment ranged from 0.3 to 2.5 min^−1^ (1.0 ± 1.0 min^−1^) for AMRB and 0.1 to 2.5 min^−1^ (0.8 ± 1.0 min^−1^) for AMSB [57]. By comparing these reported values with those obtained in this study, significant differences (*p* < 0.05) were observed between O_3_, O_3_/UV, and O_3_/UV/H_2_O_2_ treatments, which showed that O_3_/UV treatment is more effective than conventional O_3_/H_2_O_2_ treatment. These results demonstrate that O_3_-based AOPs are effective for inactivation of AMRB and AMSB in wastewater. The present findings are generally similar to the results obtained with other bacteria, pathogenic microorganisms [63,100,101], and chemical pollutants [46,49,102], thereby supporting the performance of the O_3_-based AOP for pollutants in the wastewater.

The present research established the effectiveness of O_3_-based AOP treatment for the inactivation of AMRB together with AMSB in real wastewater samples. Application of this treatment system to developing regions and countries should be encouraged worldwide for preventing the spread of infectious diseases at the stream stage. Its cost-effectiveness is also important for practical application. The use of this system to prevent the spread of infectious diseases originating from wastewater seems urgent currently. To the best of our knowledge, this is the first report to show the behaviour of AMRB and AMSB under O_3_/H_2_O_2_, O_3_/UV, and O_3_/UV/H_2_O_2_ treatments in real STP wastewater. These findings will contribute to a comprehensive understanding of the environmental risks associated with AMRB in aquatic environments.

### 3.4. Bacterial Community Structure Analysis

**Figure 3:**
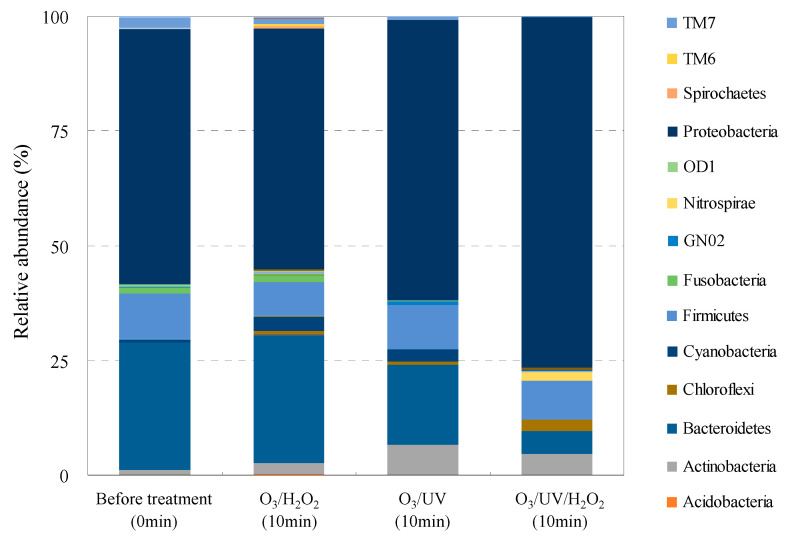
Taxonomic diversity of bacterial communities in O_3_-based AOP-treated wastewater samples.

Variations in the bacterial community structure before and after ozonation based on taxonomic affiliation of OTUs are summarized in Figure 3. The bacterial 16S rRNA reads collected from the STP wastewater samples were 142,696 at the start of treatment and 187,671 after O_3_/H_2_O_2_ treatment, 147,834 after O_3_/UV treatment, and 142,275 after O_3_/UV/H_2_O_2_ treatment (5787 OTUs in total). The STP wastewater samples hosted 40 bacterial phyla, 117 classes, 205 orders, 361 families, and 690 genera.

Interestingly, O_3_/UV and O_3_/UV/H_2_O_2_ treatment changed the constitution of phyla in the STP effluent. The constitution was Proteobacteria (55%), Bacteroidetes (28%), Firmicutes (10%), TM7 (2%), and Fusobacteria (1%) before O_3_-based treatment; Proteobacteria (61%), Bacteroidetes (17%), Firmicutes (10%), Actinobacteria (7%), and Cyanobacteria (3%) after O_3_/UV treatment; and Proteobacteria (76%), Firmicutes (8%), Bacteroidetes (5%), Actinobacteria (5%), and Chlorobi (2%) after O_3_/UV/H_2_O_2_ treatment. Meanwhile, the constitution of phyla was almost similar in O_3_/H_2_O_2_ treatment: Proteobacteria (53%), Bacteroidetes (28%), Firmicutes (7%), Cyanobacteria (3%), and Actinobacteria (2%). The genus *Acinetobacter*, comprising the major antimicrobial-resistant bacteria, also showed a reduced read rate. By comparing these results with the reports on changes in sewage flora following O_3_ treatment [57,58], it is observed that the changes in the bacterial community structure composition are almost similar for O_3_ and O_3_-based AOP treatment. The overall results suggest the importance of introducing advanced wastewater treatment for removal of AMRB and AMRGs, although some of them are not completely removed [103,104,105]. This seems reasonable considering the existence of multiple microorganisms [103,106,107] and AMRGs [108,109,110,111] in wastewater.

Recent research provides insights into the environmental risk assessment of both AMRB and AMRGs [8,35,112]. The risk of infection by AMRB in water and via the ecosystem is increasing, and further development of AMRB in the presence of residual antimicrobials or AMRGs in water is now progressing [12,113,114,115]. Further, the present study might provide invaluable information to prevent infectious diseases from the aquatic environment, including wastewater. The results improve our understanding of environmental pollution associated with AMRB and AMRGs in aquatic environments. Our findings will contribute to enhance the effectiveness of the advanced wastewater treatment systems not only at STPs but also at medical facilities such as hospitals, for reducing the discharge of AMRB and AMRGs into rivers and keeping aquatic environments safe.

### 3.5. Efficiency of the Inactivation of AMRB and AMSB Based on O_3_ Consumption

**Figure 4:**
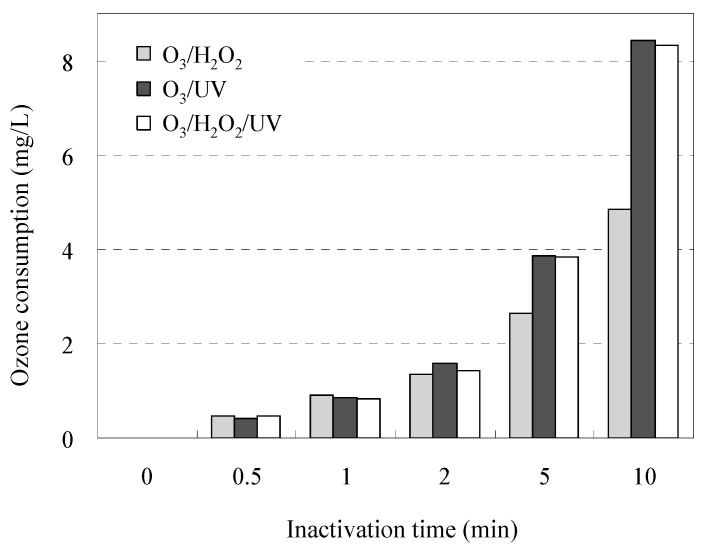
O_3_ consumption over time in STP wastewater in each treatment system.

The time-dependent profiles of O_3_ consumption during inactivation of AMRB and AMSB are shown in Figure 4. The O_3_/UV and O_3_/UV/H_2_O_2_ treatment consumed up to 1.7-fold O_3_ (1.2 ± 0.4 times by O_3_/UV and 1.2 ± 0.4 times by O_3_/UV/H_2_O_2_ as mean) compared with the O_3_/H_2_O_2_ treatment. The difference in the time-dependent profiles associated with the inactivation of AMRB and AMSB is related to O_3_ consumption by contaminants in wastewater samples during the experiment; these results agree with the distribution of reaction rate constants, as shown in Table 2. These results suggest that a long treatment process is required to achieve sufficient inactivation of a wide range of AMRB species, depending on the concentration of multiple pollutants in the wastewater.

Recent research has shed light on the environmental risk assessment of both AMRB and AMSB [8,35,112,116]. In addition, AMRB carrying antimicrobial-resistance genes (AMRGs) are also present in wastewater and river water and act as potential factors that promote the formation of new AMRB in the aquatic environment through transformations [117,118,119]. The risk of infection by AMRB in water and via the ecosystem and the development of AMRB in the presence of residual antimicrobials or AMRGs in the water environment are now being assessed [113,114,120]. Latest research reported the importance of introducing advanced wastewater treatment systems not only for wastewater at STPs but also for hospital effluents from medical facilities [30,121,122,123]. Meanwhile, it is important to maintain a balance between costs and efficiency by optimizing the operations cost of wastewater treatment along with the management of wastewater treatment plants [82,124,125]. Ozonated-fine bubble (O_3_-FB) technologies would be useful for implementing efficient and effective treatments based on O_3_ by improving the efficiency of O_3_ consumption [126,127,128]. The results should prove valuable in improving our understanding of environmental pollution caused by AMRB and AMRGs in aquatic environments. Our findings will contribute to enhance the effectiveness of the advanced wastewater treatment systems at STPs for reducing the discharge of AMRB and AMRGs into rivers, while keeping aquatic environments safe.

## 4. Conclusions

The effectiveness of inactivation induced by O_3_-based AOPs for AMRB and AMSB in STP wastewater was evaluated. The results showed that various AMRB are present in the wastewater and that O_3_-based AOPs are an effective inactivating treatment for both AMRB and AMSB. Inactivation rate constants were improved by the combined use of UV irradiation with O_3_. The estimated inactivation rate constants for AMRB and AMSB were 1.1 ± 0.8 and 1.5 ± 1.0 min^−1^ for O_3_/H_2_O_2_, 3.2 ± 2.2 and 6.4 ± 3.0 min^−1^ for O_3_/UV, and 5.6 ± 2.6 and 6.2 ± 2.6 min^−1^ for O_3_/H_2_O_2_, respectively, with half-lives generally ranging from < 0.1 to 1 min. The difference in the time-dependent profiles of inactivation for AMRB and AMSB was attributed to O_3_ consumption by contaminants in wastewater during treatment. The taxonomic diversity analysis of micro-organisms based on 16S rRNA gene sequencing showed changes in constitution of phyla after treatment, indicating that O_3_-based AOPs inactivated not only AMRB but also AMRGs present in the treated water. The overall results present a novel approach for preventing environmental risks associated with the spread of AMRB, AMSB, and infectious diseases originating from aquatic environments and contribute toward safety of water environments and human health. To the best of our knowledge, this is the first report to show the effectiveness of O_3_/H_2_O_2_, O_3_/UV, and O_3_/UV/H_2_O_2_ treatments for inactivation of AMRB and AMSB present in real STP wastewater.

## Figures and Tables

**Table 1:** Occurrence of AMRB and AMSB in sewage treatment plant (STP) influent, STP secondary effluent, and STP effluent.

Bacteria	Bacteria Counts (CFU/mL)		
	STP Influent	STP Secondary Effluent	STP Effluent
CRE	317	201	1
ESBL-E	814	182	34
MDRA	323	19	2
MDRP	98	N.D.	N.D.
MRSA	58	7	6
VRE	200	3	N.D.
*Acinetobacter*	391	38	8
*Enterococcus*	2528	836	219
*Escherichia coli*	30,000	115	N.D.
*Pseudomonas aeruginosa*	117	N.D.	N.D.
*Staphylococcus aureus*	96	8	17

CRE: carbapenem-resistant *Enterobacteriaceae*, ESBL-E: extended-spectrum *β*-lactamase-producing *Enterobacteriaceae*, MDRA: multidrug-resistant *Acinetobacter*, MDRP: multidrug-resistant *Pseudomonas aeruginosa*, MRSA: methicillin-resistant *Staphylococcus aureus*, VRE: vancomycin-resistant *Enterococcus*, and N.D.: Not detected).

**Table 2:** Reaction rate constants for each AMRB and AMSB during O_3_-based AOP treatment for STP wastewater. (* Reported values from the previous research [57].)

Bacteria	Inactivation Rate (min^−1^)			
	O_3_/H_2_O_2_	O_3_/UV	O_3_/UV/H_2_O_2_	O_3_ *
CRE	2.239	5.668	7.054	1.978
ESBL-E	0.196	3.586	3.431	0.539
MDRA	0.596	0.785	9.576	0.311
MDRP	1.290	1.976	4.242	0.523
MRSA	0.368	1.386	2.773	0.274
VRE	1.817	5.748	6.398	2.508
*Acinetobacter*	1.649	4.187	10.225	0.426
*Enterococcus*	1.165	3.496	5.776	0.725
*Escherichia coli*	2.902	9.479	4.056	2.515
*Pseudomonas aeruginosa*	1.610	9.716	6.870	0.295
*Staphylococcus aureus*	0.230	5.278	3.892	0.129

## Data Availability

Not applicable.

## Supplementary Materials

The following supporting information can be downloaded from https://www.mdpi.com/article/10.3390/antibiotics11020210/s1, Table S1: Half-life of each AMRB and AMSB during O_3_-based AOP for the model STP wastewater, Figure S1: Semi-batch ozone reactor used for experiments.
