# Supplementary material for: Inactivation of Antibiotic-Resistant Bacteria in Wastewater by Ozone-Based Advanced Water Treatment Processes
Source: Antibiotics (Basel). 2022 Feb 7;11(2):210. doi: 10.3390/antibiotics11020210 (PMC8868322; doi:10.3390/antibiotics11020210)
Supplement: Supplementary file 1 [file antibiotics-11-00210-s001.zip › antibiotics-1568148-supplementary.pdf]

Supplemental Information for

Inactivation of Antibiotic-Resistant Bacteria in Wastewater by Ozone-Based Advanced Water Treatment Processes

Takashi Azuma [1*], Masaru Usui [2], Tetsuya Hayashi [1,3]

Affiliation: Department of Pharmacy, Osaka Medical and Pharmaceutical University, Takatsuki, Osaka 569-1094, Japan

[1] Department of Pharmacy, Osaka Medical and Pharmaceutical University, Takatsuki, 569-1094, Japan; takashi.azuma@ompu.ac.jp, t.azuma.mail@gmail.com (T.A.); hayashi@gly.oups.ac.jp (T.H.)

[2] Food Microbiology and Food Safety, Department of Health and Environmental Sciences, School of Veterinary Medicine, Rakuno Gakuen University, Ebetsu, 069-8501, Japan; usuima@rakuno.ac.jp

[3] Faculty of Human Development, Department of Food and Nutrition Management Studies, Soai University, Suminoe-ku, 559-0033, Japan; t.hayashi@soai.ac.jp

*Corresponding author: Takashi Azuma

Affiliation: Department of Pharmacy, Osaka Medical and Pharmaceutical University, Takatsuki, 569-1094, Japan

Tel: +81-72-690-1055, Fax: +81-72-690-1055

e-mail address: takashi.azuma@ompu.ac.jp, t.azuma.mail@gmail.com

Table S1. Half-life of each AMRB and AMSB during O3 based AOP treatment for the model STP wastewater.

Bacteria	Half-life (min)			
	O_3/H_2O_2	O_3/UV	$O_3/UV/H_2O_2$	O_3^*
CRE	0.3	0.1	0.1	0.4
ESBL-E	3.5	0.2	0.2	1.3
MDRA	1.2	0.9	0.1	2.2
MDRP	0.5	0.4	0.2	1.3
MRSA	1.9	0.5	0.2	2.5
VRE	0.4	0.1	0.1	0.3
Acinetobacter	0.4	0.2	0.1	1.6
Enterococcus	0.6	0.2	0.1	1.0
Escherichia coli	0.2	0.1	0.2	0.3
Pseudomonas aeruginosa	0.4	0.1	0.1	2.4
Staphylococcus aureus	3.0	0.1	0.2	5.4

*Reported values from the previous research [57].

Values for the model STP wastewater prepared by mixing STP influent and STP secondary effluent (1:9 (v/v). CRE: carbapenem-resistant *Enterobacteriaceae*; ESBL-E: extended-spectrum *β*-lactamase-producing *Enterobacteriaceae*; MDRA: multi-drug-resistant *Acinetobacter*; MDRP: multi-drug-resistant *Pseudomonas aeruginosa*; MRSA: methicillin-resistant *Staphylococcus aureus*; and VRE: vancomycin-resistant *Enterococcus*).

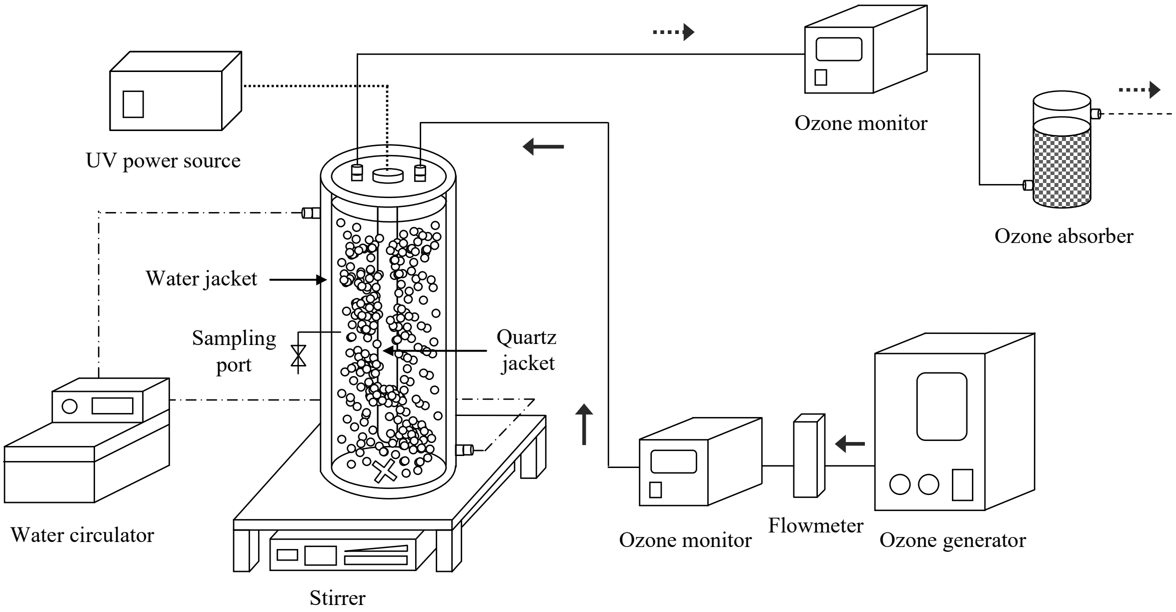

Figure S1. Semi-batch ozone reactor used for experiments.
